# Supplementary material for: Identification of an Acidic Amino Acid Permease Involved in d-Aspartate Uptake in the Yeast Cryptococcus humicola
Source: Microorganisms. 2021 Jan 18;9(1):192. doi: 10.3390/microorganisms9010192 (PMC7831105; doi:10.3390/microorganisms9010192)
Supplement: Supplementary file 1 [file microorganisms-09-00192-s001.pdf]

Supporting information

Identification of an acidic amino acid permease involved in D-aspartate uptake in the yeast *Cryptococcus humicola*

Daiki Imanishi [1], Yoshio Kera [1] and Shouji Takahashi [1,*]

[1] Department of Bioengineering, Nagaoka University of Technology, Nagaoka, Niigata, 940-2188, Japan.

* Correspondence: shoutaka@vos.nagaokaut.ac.jp; Tel: +81 258 47 9667

Table contents

Figure S1. Comparison of amino acid sequences of ChAap4 and ChAap5 with AdiC, Dip5p, PcDip5, and AgtA.......... **2**

Figure S2. Exon-intron structures of *ChAAP4*, *ChAAP5*, and acidic Aap genes.......... **4**

Table S1. Primers used in this study **5**

```
AdiC    1   MSSDAD------------------------------------------------------------------------------AHK-VG 11
ChAap4  1   MSGYNNYNN--GNN----------------------------EYKSELGPYEGGN-EKALATDHVYAAQDDYHVD--AEPQQEETHRSLK 57
ChAap5  1   MAKDYESDS--------------------------------------PGPYEGSN-EKALGHDFT-TQVDDGSTN--DPPHQEETHRLLK 48
Dip5p   1   MKMPLKKMFTSTSPRNSSSLDSDHDAYYSKQNPDNFPVKEQEIYNID---LEENNVSSRSSTSTSPSARDDSFAVPDGKDENTRLRKDLK 87
PcDip5  1   MGHPDEINALEAQP----------------------------GY--------------------V-DNLGDFKES--EQDEPVQLHRELK 39
AgtA    1   MGVEKDDGITA-APQN----------------P---------VY--------------------D-ASVAEKGVL--EVPQENELHRALK 41
            *    .                                                                                :: :

                TM1 ●●●                     TM2                                         TM3
AdiC    12  LIPVTLMVSGNIMGSGVFLLPANLA-STGGIAIY-GWLVTIIGALGLSMVYAKMSFLDPSPGGSYAYARRCFGPFLGYQTNVLYWLACWI 99
ChAap4  58  PRQISMIAIGGAIGTGLVIGSGTSLIRSGPGSLFLSYVIMGFCCFSVMIALGEMSTKFPSKKGFAGHATRCVDPAFGFATALVYLCKYLI 147
ChAap5  49  PRQISMIAIGGAIGAGFLIGSGTSLARSGPASMLIAYICMGFCCFAVMIALGEMSTKFPTKKGFSGHATRCVDPAFGFATALIYLCKYLI 138
Dip5p   88  ARHISMIAIGGSLGTGLLIGTGTALLTGGPVAMLIAYAFVGLLVFYTMACLGEMASYIPL-DGFTSYASRYVDPALGFAIGYTYLFKYFI 176
PcDip5  40  ARHISMIAIGGAIGTGLVVGTGTSAL-TAGPGSLFIAYCFMGFVVWTVMCGLGEMAAWLPLSSGFTGYAGRFCDPALGFTLGWCYYLKYII 128
AgtA    42  ARHITMIAIGGAIGTGLIIGTGSALEKAGPGAILISYAFMGFIVYLVMCGLGEMATWLPRSSGFTGYAVRFCDPALGFALGYTYWFKYII 131
               ::::. *. :*:*..: ...     *  ::  .:    :          .:*:   *   *  .:* *  .* :*:      *     *

                                            TM4                           TM5
AdiC    100 GNIAMVVIGVGYLSYFF----PILKDPLVLTITCVVVLWIFVLLNIVGPKMITRVQAVATVLALIPIVGIAVFGW--------------- 170
ChAap4  148 ISPNQIVAGALVIRFWN----AKINGAAWVTIL----VALVIALNLLGIKWFGEVEFWLSLLKVVTLTGLIILGLIIDLGGVPGQERIGF 229
ChAap5  139 ISPNQLVAGALMIRYWS----HDINGAVWVTIL----GALVFAINFLGVKWFGEVEFWLSLFKITVLTGLIILGLIIDLGGVPGQDRIGF 220
Dip5p   177 LPPNQLTAAALVIQYWIS--RDRVNPGVWITIF----LVVIVAINVVGVKFFGEFEFWLSSFKVMVMLGLILLLFIIMLGGGPNHDRLGF 260
PcDip5  129 LPPTQLTAAALVITYWPRTSAENVNPGVWIAIF----MVAIIVINYFGVKIFGELEFWLSSFKVIVILGLILCSLLFALGGGPDHDRRGF 214
AgtA    132 VTPNQLTAAALVIQYWLP--ADKVNPGVWITVF----LVLIVFINYFGVGFFGEFEFWLSSFKVVVIVGLILLSFILMLGGGPDHDRKGF 215
                 :. ..  : ::       ::    :::       .. :* .*    :  ..:    : : :   : *: :

                                       TM6 ▼▼▼▼▼▼▼                                   TM7
AdiC    170 -FWFRGETYMAAWN------VSGLGTFGAIQSTLNVTLWSFIGVESASVAAGVVKNPKRNVPIATIGGVLIAAVCYVLSTTAIMGMIPN- 252
ChAap4  230 RYWKDGRAFKPYKQ------PGDLGKFLGFVNALILALFAYMGTELVGVTVGEAKNPRKTVPSAIRKTFYRILFFYILGT-LIVGMIVDS 312
ChAap5  221 RYWKK-AAFKSYKA------PGKTGQFLGFVNALVLALLAFMGSELIGMTVGEAKNPRETIPSAVKKTFFRISFFFVFGT-FVVGLVADS 302
Dip5p   261 RYWRDPGAFKEYSTA----ITGGKGKFVSFVAVFVYSLFSYTGIELTGIVCSEAENPRKSVPKAIKLTVYRIIVFYLCTV-FLLGMCVAY 345
PcDip5  215 RYWNKPGAFATKYT------DGALGKFLAFWSTMVQATFAFLGTELIGVTVGEAQNPRKTIPKAIKLTFWRIIVFYILSV-LFLGMLVPY 297
AgtA    216 RYWKDPGAFNSYIDMGPDGNGSAAGRFYAFWATLVSATFAYLGTELVGVTVGEAQNPRKTIPRAIKLTFYRIIFFYVISV-LLVGTLVPY 304
             :*    ::               .  * * .:  .:  :  :: * *  .:. . .:**:..:* *      .    . ::  .  .:*

                        ◆           ◆          TM8                                           TM9
AdiC    252 ------------AALRVSASPFGDAARMALGDTAGAIVSFCAAAGCLGSLGGWTLLAGQTAKAAADDGLFPPIFARVNKAGTPVAGLIIV 330
ChAap4  313 NDPLLAAAAKKGTSGGAAASPYVVAIQSAKIKVLPHIINACILMFTISAANSDQYIASRTLYGMAMDGNAPRIFRYCTKRGVPMAAFIAT 402
ChAap5  303 TSDLLATAA-KNSHGSSSASPWVVAIKLANIKVLPGIINGCLLVYTISAAVSDQYISSRTLYGMAMDGHAPAIFKKCTKRGVPITAFAFT 391
Dip5p   346 NDPRLLST--KGKSMSAAASPFVVAIQNSGIEVLPHIFNACVLVFVFSACNSDLYVSSRNLYALAIDGKAPKIFAKTSRWGVPYNALILS 433
PcDip5  298 DSKELAFA--TEAESSAAASPFVVAM--GQVAALPHIINGCILVFVFSAANSDLYIATRTLYGLAREKKAPKIFARTNRAGVPIYSLALS 383
AgtA    305 DSSELKQA--NSASTSSAASPFVLAIQLSGIPALPHILNACILAFVFSAANSDLYIATRTIYGLAKEGKAPKILTKTDRRGVPFVALGLC 392
                             :***:  *    .    .    *.. *     :.:   .    :: :.   . * :    * *:     : *.*   .:

                               ◆           TM10                                     TM11
AdiC    331 GILMTIFQLSSISPNATKEFGLVSSVSVIFTLVPYLYTCAALLLLGHGHFGKARPA--------------YLAVTTIAFLYCIWAVVGSG 406
ChAap4  403 GMFMGLAYLVA-AESALTVFNYFVNSVSVFGGLAWIS-----ILASHIGFTRGMRAQGIPRDTLPYRSSLQPYLSYVALPLVCIIIFFKG 486
ChAap5  392 GAFMGLAYLVA-ADGALVIFNYFVNSVTICGGLSWIS-----ILSSHIAFMRGHKAQGIPRSELPFAFQCQPYMSYIALTMVSILMFFKG 475
Dip5p   434 VLFCGLAYMNV-SSGSAKIFNYFVNVVSMFGILSWIT-----ILIVYIYFDKACRAQGIDKSKFAYVAPGQRYGAYFALFFCILIALIKN 517
PcDip5  384 ASFCLLAFMSV-SSTSKEVFGYFTDMVSIFGLLTWVS-----LLITHIFFIRARRAQGVDQATLAYKAPLGIIGSCIALFFCVLVLFTRS 467
AgtA    393 TLIALIAYMNV-SSSSKTVFKYFVDLVSQFGLLSWIS-----LLVTHICFVKARKAQEIPKNELAYTAPLGVWGSYFALFWCIVVSLTKS 476
               :  :  :   :  :    *  . .          :.::        :*  :  * :.  *                    :  .*:       .  .

                          TM12
AdiC    406 ------A-----KEVMWSFVTLMVITAMYALN---YNRLHKNPY--PLDAPI--SKD--------------------------------- 445
ChAap4  487 FDSFMPMS-AFGKTGYKTFITHYIGIPVYFFGYVGFKFIRRTSYVRLHEMDLNSGAREFHDVDDDDEEDERYKQLSFGEKIKHNLK--NW 573
ChAap5  476 FDSFVPIS-QFTTTGYKTFITHYIGVPVYIFGYFGYKFAHKTKVVRLDTMDLKAGGREFHDVVEDDEEERAYKAMSVKGKIAYQLK--NW 562
Dip5p   518 FTVFLGH-----KFDYKTFITGYIGLPVYIISWAGYKLIYKTKVIKSTDVDLYTFK----EIYDREEEEGRMKDQEKEERLKSNGKNMEW 598
PcDip5  468 FGVFIHDPETYGNFDYKTFITSYIGIPLYVAAFAGWKIWNKTEIIKPHNADIWTGK----AEIDREEAEYVAMV----AIQDQNLSGWNK 549
AgtA    477 FDVFTGD-----TFNYKTFITSYLGIPLYLILIFGYKFTTRCKRVLPHEADLYTDK----DAIDREEAAYLARQEAKRAAQGANHKQANW 557
                         .    :*:*  :  .:*       ::   :           :

AdiC    445 ---------- 445
ChAap4  573 ---------- 573
ChAap5  562 ---------- 562
Dip5p   599 FYEKFLGNIF 608
PcDip5  550 VYRKSLAWLF 559
AgtA    558 FYKTFVSWLF 567
```

Figure S1. Comparison of amino acid sequences of ChAap4 and ChAap5 with *E. coli* AdiC (UniProKB: P60061) and three yeast dicarboxylic amino acid permeases: Dip5p (*S. cerevisiae*), PcDip5 (*P. chrysogenum*), and AgtA (*A. nidulans*). Transmembrane (TM) regions were predicted by Phobius (http://phobius.sbc.su.se/) and boxed in black. GXG and (F/Y)(S/A/T)(F/Y)XGXE motifs (where X is any amino acid) are indicated by closed circles and triangles, respectively. The

amino acid residues highly conserved in dicarboxylic acid transporters are indicated by closed diamonds [27].

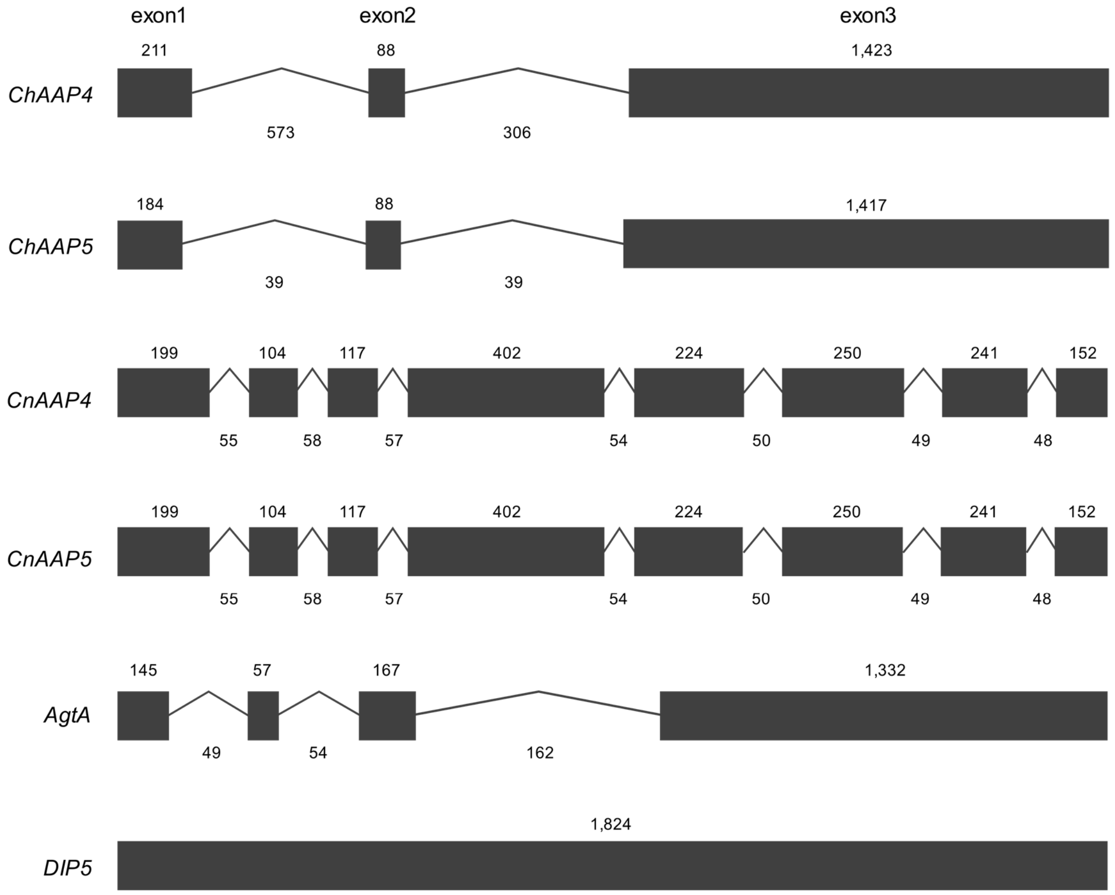

Figure S2. Exon-intron structures of *ChAAP4*, *ChAAP5*, and acidic Aap genes. The closed box and thine line indicate exon and intron, respectively, and the number indicates the size (bp). The scale ratio of exon and intron is not constant. The full-length genomic DNA of *ChAAP4* gene is 2,601 bp with an CDS of 1,719 bp. The full-length genomic DNA of *ChAAP5* gene is 1,767 bp with an CDS of 1,686 bp. The nucleotide sequence identity between exon1, exon2, and exon3 of *ChAAP4* and *ChAAP5* genes was 62.7%, 71.6%, and 72.1%, respectively.

Table S1. Primers used in this study.

Primers	Sequence (5′ to 3′)
For constructing gene-disrupting cassettes	
URA3F	CGAGGTCGACGGTATCG
URA3R	CAGGAAACAGCTATGAC
AAP4UF	CGCGGCATCACGTGAAGTAC
AAP4UR	CGATACCGTCGACCTCGTCCTGGGCTGCGTAGACATG
AAP4DF	GTCATAGCTGTTTCCTGATCAGTGGCGACGAGACGAG
AAP4DR	CACCGGCGACAATCTGGTTG
AAP5UF	TCGAGCCGCTCTTCAGTACG
AAP5UR	CGATACCGTCGACCTCGGCACGCCACCGAGATCAATG
AAP5DF	GTCATAGCTGTTTCCTGTCCCACATTGCGTTCATGCG
For checking gene disruption	
4UFwd1	ATCTGGACCGACACAGGTAG
4URev1	TTGCAGGGTCGAATGGAATG
4DFwd2	ACTTTCTGCGCGTGGTAAAC
4DRev2	GTACCCATGTAGGCGAAGAG

5UFwd2	CTCTGGTTCACCGAGAGGAG
5URev2	TCTCTTCAGCCTTGGGAGTC
5DFwd3	CCTTGTGCTGCTCTTGATTG
5DRev3	TCACGATCGTCTTCCAGATG
For qRT-PCR	
RTChDDOF2	CTCACGCAGACCGAGATGTG
RTChDDOR2	GTCGAGCACGCGGAAATCT
RTChTAF10F	CGAGGAGGTGACCGAGTACT
RTChTAF10R	CAGAGACAACAGGCGCTTTAGTC
